# From primal scenes to synthetic cells

**DOI:** 10.7554/eLife.46518

**Published:** 2019-03-13

**Authors:** Hub Zwart

**Affiliations:** Erasmus School of PhilosophyErasmus University RotterdamRotterdamThe Netherlands

**Keywords:** History of Science, Philosophy of Biology, Cell Biology, Synthetic Cells

## Abstract

Synthetic cells spark intriguing questions about the nature of life. Projects such as BaSyC (Building a Synthetic Cell) aim to build an entity that mimics how living cells work. But what kind of entity would a synthetic cell really be? I assess this question from a philosophical perspective, and show how early fictional narratives of artificial life – such as the laboratory scene in Goethe’s *Faust* – can help us to understand the challenges faced by synthetic biology researchers.

The idea of producing something life-like in vitro – or, more precisely, the idea of building a synthetic cell – has been a key objective of synthetic biology from the very outset of the field. Over the past twelve decades or so, living cells have been effectively dissolved into their basic molecular and informational components. Now the time has come to put all these components together again, in order to address the question: do we really understand life, or have we missed something?

I am a principal investigator on BaSyC, a project that aims to use basic components to build a synthetic cell that convincingly mimics all the functions of a living cell. BaSyC takes an engineering approach to this challenge, starting from the conviction that we can only genuinely claim to understand life insofar as we can technologically reproduce it. In other words, to effectively demonstrate that the essence of life has been duly grasped, we should be able to produce something in vitro which not only maintains its internal metabolism but can actually reproduce itself.

As a philosopher in science, I study scientific endeavours from an oblique or sideways perspective (Zwart, 2017; Zwart, 2018). This means that my focus of attention concerns both the object pole (that is, the synthetic cell) and the subject pole of the knowledge production process (the scientists and research groups working on this challenge). In other words, my focus is not on the technical or compositional features of synthetic cells, but on the dynamical interplay between subject and object.

My oblique perspective consists of three basic methodological components. First of all, it builds on the conviction that, in order to understand the present and the emerging future, we have to take a step backwards in time and return to the primal scene of synthetic biology research: the birth of the desire to create life in a test tube. A second methodological principle is triangulation, which means that we need a third position from which we can attempt to unravel the interaction between the subject and the object. Such a position may be provided by novels, movies or plays about the desire to create artificial life. Thirdly, a synthetic cell would be a new type of thing – an ontological novelty in the language of philosophy – and, therefore, highly likely to lead to a range of responses.

Synthetic cells will trigger both fascination and unease, and it is in works of the imagination – such as novels and plays – that we find these mixed responses articulated, addressed and fleshed out, albeit often in a dramatized fashion. This explains why, through critical readings of science novels, science cinema and science theatre, we may explore (and to some extent predict) how novelties such as synthetic cells will be received by public audiences.

## The birth of the idea of creating life in vitro

Several decades before synthetic biology – as we currently know it – got off the ground, the signifier ‘synthetic biology’ had already been coined by Stéphane Leduc (in 1912) to refer to his research, which involved growing crystals in solutions to mimic and explore organic forms (Campos, 2009). Even earlier, in 1905, the physicist John Butler Burke was exposing petri dishes containing bouillon to radium to produce artificial life-like forms, inspired by his conviction that radium could generate life. These and similar experiments conveyed the idea, articulated by the biologist Jacques Loeb and others, that 20th century biology should become biotechnology (Pauly, 1987). As JBS Haldane said, paraphrasing Marx: “the vocation of scientists is not to explain the living world, but to change it” (Haldane, 1939). However, it was only with the discovery of recombinant DNA techniques in the 1970s that the engineering approach to biology, also known as ‘intentional biology’, really took off (Campos, 2009).

The idea of creating life in a test-tube had thrived in books and plays long before it became real science.

Yet, the idea of creating life in a test-tube had thrived in books and plays long before it became real science. Goethe (1831) explored the idea of creating life in a laboratory in his drama *Faust*, in a scene entitled Laboratory (Part II Act II Scene 2; 6819 ff.), in which Faust’s trusted disciple Wagner successfully manages to fabricate an artificial ‘little man’ (a homunculus) in a glass phial in his oven. Goethe’s scene became a classical enactment of 'Faustian’ science (Spengler, 1918): a form of science bent on controlling and enhancing nature, rather than on understanding it.

As indicated above, a rereading of this ‘primal scene’ may enlighten some of the expectations and concerns raised by synthetic biology, and, more precisely, by synthetic cell projects. Inside his phial, which he carefully keeps at the right temperature, Wagner has composed the right mixture with the right components to create a kind of primal soup and, exactly at the right moment (when the bell tolls), he discerns the budding signs of neo-life. Something is glowing inside the tube: a luminous substance, reminiscent perhaps of the time-old association between life and light that was taken up by the physicist Niels Bohr in his famous lecture *Light and Life* (Bohr, 1933). After many detours and faux pas, Wagner’s hazardous experiment seems about to succeed at last. What nature brings forth in vivo can now ‘crystallize’ inside a glass tube. Artificial life has been created in the lab.

What Wagner fabricated, I would argue, was not so much an artificial life form as a prolific narrative script, one that would soon proliferate through the works of many other authors: from Mary Shelley (*Frankenstein*) via HG Wells (*The Stolen Bacillus*) up to contemporary writers. Indeed, some of these themes had already been fleshed out quite recognisably by Goethe in *Faust*. To begin with, he stresses the fragility and vulnerability of neo-life. The homunculus can only survive inside his tube – in a gnotobiotic environment, technically speaking. Should the glass phial break, exposure to a normal environment would prove fatal. This is a reassuring thought, suggesting that neo-life can, in the modern language of biosafety and biosecurity, be ‘contained’. The survival of the artefact depends on qualified researchers and the controlled environments that they create. Neo-life is and will remain a laboratory artefact.

Unfortunately, this reassuring impression does not last because, at precisely this moment, when Wagner is about to finish his great experiment, there is a knock on the door and Mephistopheles enters the room with the explicit intention of stealing the tube. Mephistopheles flees the laboratory with the homunculus hidden under his cloak, leaving Wagner behind to resume and improve his experimental work. In the current literature, this aspect is discussed under headings such as ‘biosecurity’ or ‘dual use’. Scientists may conduct their hard work with the best of intentions, but others may use the results of this work for nefarious goals. In this manner, with the help of others, fragile little ‘monsters’ may escape the laboratory setting after all.

In other words, a question emerges as soon as we are able to control life: how do we control this power to control life? And if life can be technologically domesticated, how do we domesticate this new and powerful technology itself? A similar question currently harrows the discoverers of CRISPR-Cas9 (Doudna and Sternberg, 2017). To address this question, our third methodological principle must be activated, and the ontological dimension of synthetic life must be addressed. What will we be dealing with?

## What kind of thing is a synthetic cell?

There seems to be something exceptional about creating synthetic life compared to other laboratory fabrications. The theory of vitalism proposes that life is governed by a force distinct from the standard chemical and physical forces that act on inanimate objects. In 1944, the physicist Erwin Schrödinger portrayed life in terms of deviation: whereas nature is under the sway of entropy, living cells maintain high levels of complexity and resist disruption. (Back in 1933, Niels Bohr had also pointed out how living systems seem to defy the expectations of physics and chemistry.) Schrödinger argued that the essence of life is not Divine intervention (Genesis), electricity (Frankenstein) or radium (Burke), but a genetic code: the genome (for comparison, see Zwart, 2013).

A question emerges as soon as we are able to control life: how do we control this power to control life?

The discovery of the molecular structure of DNA by Watson and Crick was perceived by many as a fatal blow to vitalism, for the genome is basically a molecule: an “aperiodic crystal”, as Schrödinger phrased it. Once we understand this crystal, we will eventually be able to (in Wagner’s terms) ‘crystallize’ life in vitro.

Does this mean that public concerns should be discarded as outdated remnants of vitalism? Again, Goethe’s scene provides a telling hint, for Wagner explicitly points out that his current experiment is merely a first step. Eventually, the idea is not to create life as such, but to produce something even more ambitious and potentially more disconcerting: something like a thinking brain, challenging our unique position as rational entities. However fragile and innocent synthetic cells may seem, public discontent is triggered by the suspicion that this is merely a first step towards something more challenging and prolific: a second Cambrian explosion of life-forms may await us (Church and Regis, 2013). How will these entities evolve? And do we have the tools and policies in place to govern and contain this potentially disruptive process?

As to the question “What kind of thing is a synthetic cell?”, the famous article *What is a thing?* by the philosopher Martin Heidegger provides a route to an answer (Heidegger, 1954). In it, he explores the etymology of the term “thing”, connecting it with the Old High German “thing” or “dinc” and the proto Germanic “*þingą”*, which mean an assembly or congregation. A synthetic cell is *a thing which calls for a Thing*: a public deliberation. In 1974, a panel established by the National Academy of Sciences in the US argued for a thorough assessment of the potential social impacts of recombinant DNA techniques (Berg et al., 1974). A similar assessment, based on multiple voices and perspectives, is now needed to explore the potential impacts of synthetic cells.

## Note

This Feature Article is part of the Philosophy of Biology collection.
